# The paradox of a long grounding during West Antarctic Ice Sheet retreat in Ross Sea

**DOI:** 10.1038/s41598-017-01329-8

**Published:** 2017-04-28

**Authors:** Philip J. Bart, Benjamin J. Krogmeier, Manon P. Bart, Slawek Tulaczyk

**Affiliations:** 1 Department of Geology and Geophysics, Louisiana State University Baton Rouge, Baton Rouge, LA 70803 USA; 2 Department of Earth and Planetary Sciences, University of California Santa Cruz, Santa Cruz, CA 95064 USA

## Abstract

Marine geological data show that the West Antarctic Ice Sheet (WAIS) advanced to the eastern Ross Sea shelf edge during the Last Glacial Maximum (LGM) and eventually retreated ~1000 km to the current grounding-line position on the inner shelf. During the early deglacial, the WAIS deposited a voluminous stack of overlapping grounding zone wedges (GZWs) on the outer shelf of the Whales Deep Basin. The large sediment volume of the GZW cluster suggests that the grounding-line position of the paleo-Bindschadler Ice Stream was relatively stationary for a significant time interval. We used an upper bound estimate of paleo-sediment flux to investigate the lower bound duration over which the ice stream would have deposited sediment to account for the GZW volume. Our calculations show that the cluster represents more than three millennia of ice-stream sedimentation. This long duration grounding was probably facilitated by rapid GZW growth. The subsequent punctuated large-distance (~200 km) grounding-line retreat may have been a highly non-linear ice sheet response to relatively continuous external forcing such as gradual climate warming or sea-level rise. These findings indicate that reliable predictions of future WAIS retreat may require incorporation of realistic calculations of sediment erosion, transport and deposition.

## Introduction

**Figure 1:**
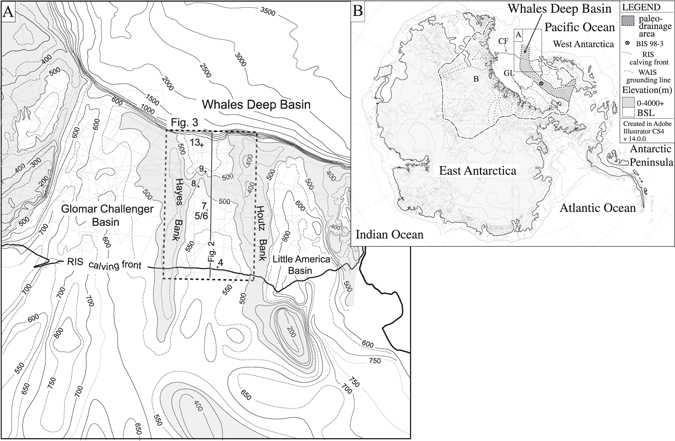
(**A**) Bathymetry of eastern Ross Sea^7, 8^ (CI = 50 m). The light-gray rectilinear grid shows the seismic grid. The bold line shows the location of the seismic line shown in Fig. 2. The dashed box shows the location of Fig. 3. (**B**) The dark gray shade shows the drainage area for the paleo-Bindschadler Ice Stream when grounded ice advanced to the outer shelf^9, 10^ superimposed on an elevation map of Antarctica^11^. The circled × marks the location of borehole Site 98–3^2^. The long- and short-dashed lines show ice sheet drainage and sub-drainage divides for Ross Sea. RIS = Ross Ice Shelf; GL = Grounding line; CF = Calving front. B = Byrd glacier drainage area.

The Antarctic continent is near completely covered by grounded ice with large expanses of floating ice extending into marine embayments (Fig. 1). The Ross Embayment is the largest catchment area (i.e., drainage basin) receiving ~25% of total Antarctic ice flow^1^ (Fig. 1b). The slow flow of grounded ice in the interior regions accelerates into ice streams and rapidly transfers grounded ice to the floating Ross Ice Shelf. Rapid ice stream flow is accomplished by till deformation and/or basal sliding^2^. The overall ice sheet mass balance between snow accumulation and ice mass loss through iceberg calving as well as surface and basal melting affects global sea level.

During the most recent glacial cycles of the Pleistocene, grounded ice advanced and retreated over a distance of more than 1000 km from the Ross Sea inner shelf to the continental shelf edge and back^3^. On the outer continental shelf, the large-scale bathymetric troughs and banks provide compelling evidence that six erosive ice streams drained the Pacific sector of the WAIS by the end of the last glacial cycle (Fig. 1a). During the peak of the LGM, sediment eroded from the Ross Sea catchment was transported in basal debris zones and deposited at the marine terminus of ice streams at either the outer shelf or upper slope^3–5^. As grounded ice retreated during the subsequent deglaciation, the paleo-ice-stream troughs were partly backfilled with trough-confined GZWs. These wedges are subaqueous sediment accumulations formed at the mouth of ice streams when the grounding line is stationary or slightly advancing. In dip view, these deposits are asymmetrical low-relief features with a gentle landward dipping topset and a more steeply basinward dipping foreset. These GZWs are important because they provide unequivocal evidence as to the former locations at which the ice sheet paused during its overall retreat. In addition, GZWs may help protect grounded ice from retreat forced by sea level rise^6^.

Sediment from shallow-penetration piston and kasten cores recovered from the Ross Sea continental shelf grade upwards from tills deposited by or in proximity to grounded ice to an overlying condensed section (a few tens of centimeters thickness) of post-glacial sediment deposited after the retreat of grounded ice^5^. Precisely dating ice sheet advance and retreat is challenging because the proximal glacial settings contain very little carbonate and total organic content is low^4, 5^. The existing strategy is to date the resumption of marine sedimentation that follows the retreat of grounded ice^4, 5^. Previous studies confirm a LGM age for the last advance of the WAIS and a post-LGM age for the ongoing retreat^3^. Despite the consensus concerning this general chronology^3–5^, it has not yet been possible to constrain the duration of individual groundings (required by stratigraphic superposition of backstepped GZWs) because the current number of core sites with good-quality radiocarbon dates is too coarsely spaced along the axes of the paleo-trough systems^3–5^. Hence, the interval of time when ice sheet paused at any one location during the ongoing post-LGM retreat remains unknown.

In eastern Ross Sea, the bathymetric saddle on the outer shelf of the Whales Deep Basin^7^ (Fig. 1) is a particularly large-volume compound GZW (CGZW)^4^. The wedge was deposited by the paleo-Bindschadler Ice Stream of the WAIS (Fig. 1a,b)^3, 7–11^. Here, we used a new approach to estimate the Whales Deep outer-shelf grounding duration (Fig. 1) that does not rely on radiocarbon dates. The new strategy improves upon a previous approach^12^, which was used to estimate the duration of a grounding event on the middle shelf of the Glomar Challenger Basin paleo-ice-stream trough (Fig. 1).

## Methods

Our study of the Whales Deep outer-shelf grounding duration involved three distinct objectives. The first objective concerned mapping the CGZW extent by seismic correlation. We used high-resolution single- and multi-channel seismic profiles from six surveys that include strike and dip profiles (Fig. 1). The interpreted seismic profiles were used to create an isopach contour map showing the CGZW thickness in milliseconds of two-way travel time. The contour map was converted to depth and then used to calculate the CGZW volume using a sediment velocity of 1,750 ms^−1^ based on seismic-derived velocity estimates of high-latitude glacigenic sediments^13^.

**Figure 2:**
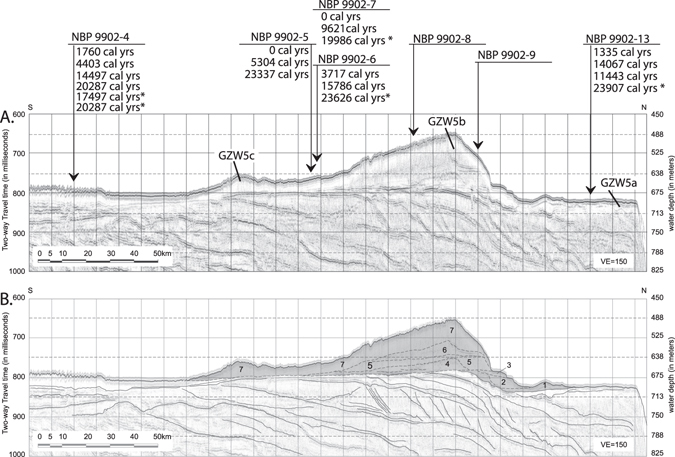
(**A,B**) Uninterpreted and interpreted dip-oriented multichannel seismic line 1502B-03 along the axis of Whales Deep from the shelf edge to within ~10 km of the Ross Ice Shelf calving front (see Fig. 1 for location). Core locations and corrected radiocarbon dates from Mosola and Anderson^4^ are from acid insoluble organic matter within sediments deposited since the retreat of grounded ice. The gray-shaded area shows dimensions of the composite GZW corresponding to the location of the bathymetric saddle at the outer shelf. The GZWs are labeled 1–7 from oldest to youngest but it is not possible to map the individual lobes because the top and base reflectors are not continuous across the study area.

The second objective involved generating an empirical estimate of paleo sediment yield (*S*_p_) for the Whales Deep Basin paleo-ice-stream trough. The estimate of paleo sediment yield was applied to the larger drainage basin area that existed when the WAIS was grounded on the outer continental shelf (Fig. 2) to calculate a paleo sediment flux (*Q*_P_) for the Whales Deep paleo ice stream trough using Equation (1). Data summarized by Elverhoi *et al*.^14^ indicate that glacial erosion rates tend to be independent of drainage basin size. Hence, our upper bound estimate for paleo sediment yield was applied to the larger drainage basin area that existed when the WAIS was grounded on the outer continental shelf (Fig. 2) to calculate a paleo sediment flux (*Q*_P_) for the Whales Deep paleo ice stream trough using Equation (1).

1 $${Q}_{{\rm{p}}}({m}^{3}{a}^{-1})=Drainage\,are{a}_{{\rm{p}}}({m}^{2})\times {S}_{p}({m}^{3}{m}^{-2}{a}^{-1})$$

In the third part of our study, we used the paleo sediment flux (*Q*_Paleo_) to estimate the duration of the outer-shelf grounding events using Equation (2):

2 $$d={v}_{s}/{Q}_{{\rm{p}}}$$

where *d* is duration in years (a), *v*_*s*_ is CGZW sediment volume (in m^3^), and *Q*_P_ is the Whales Deep paleo-ice-stream sediment flux (in m^3^a^−1^).

## Results

### CGWZ seismic stratigraphy and sediment volume (*v*_*s*_)

Stratigraphic superposition via seismic correlations show that the Whales Deep CGZW overlies a regional unconformity that can be projected as a seafloor reflection at the shelf edge into the subsurface across the outer- and the middle-shelf portions of the Whales Deep trough (Fig. 2). On the outer continental shelf, the CGZW overlying this unconformity is composed of seven trough-confined stacked GZWs. The individual wedges are labeled GZW 1 through 7 from oldest to youngest. The first three GZWs are comparatively thin (<25 msec two way travel time (TWTT, i.e., 20 m) and have shorter extents on dip-oriented profiles but the top and base reflectors cannot be seismically resolved across the study area (Fig. 2). Seismic correlations show that there are no seismically resolvable GZWs in the area south of bathymetric saddle and north of the modern Ross Ice Shelf calving front (Fig. 2).

**Figure 3:**
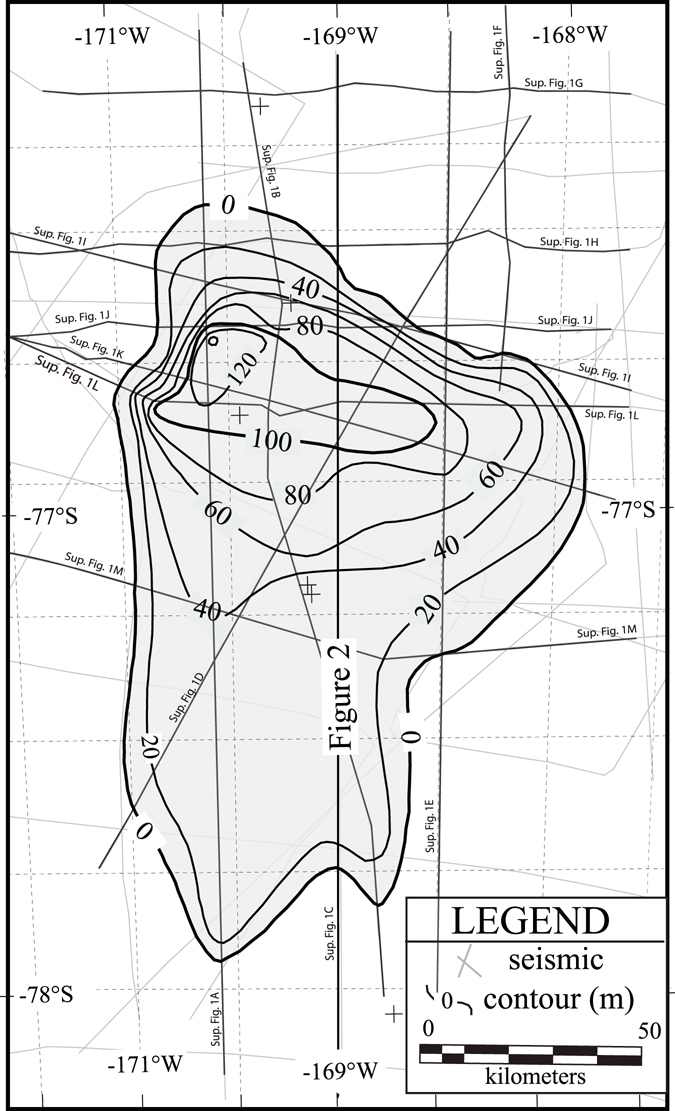
Isopach map of the composite GZW in meters. The contour interval is 10 m. The light-shaded rectilinear grid shows the location of seismic profiles used to map the distribution of the unit.

The correlations on strike- and dip-oriented seismic transects (Supplemental Figure 1) show that the CGZW has a large thickness distributed over the central part of Whales Deep Basin between the flanks of the Hayes and Houtz Banks (Fig. 3). The isopach map (Fig. 3) highlights that the CGZW is trough confined and thickens gradually from the middle shelf to a tight east-west trend of contours at the bathymetric saddle. The cluster has a maximum thickness of 140 m at the crest of the saddle in the axes of the trough and thins abruptly in a basinward direction to a depositional pinchout limit on the outer shelf. Volumetric analysis of the isopach map reveals that the CGZW contains a sediment volume of 5.34 ± 0.25 × 10^11^ m^3^ (Table 1).

**Table 1:** (A) Low and high modern Antarctic sediment yield estimates from Alley *et al*.^19^ and paleo sediment yield; (B) Modern and paleo Bindschadler Ice Stream drainage area; (C) Sediment flux for the modern and paleo Bindschadler Ice Stream; (D) CGZW volume calculated in this study; (E) duration of WAIS grounding on the outer continental shelf of the Whales Deep Basin.

Location	A. Yield *S* (m^3^m^−2^a^−1^)	B. Drainage Area (m^2^)	C. Sediment Flux *Q* (m^3^a^−1^)	D. CGZW Volume (m^3^)	E. Duration (a)
Modern grounding line	0.4 × 10^−3^ 0.5 × 10^−3^	1.23 × 10^11^	4.92 × 10^7^ 6.15 × 10^7^	—	—
Paleo grounding line	0.62 × 10^−3^	2.32 × 10^11^	1.42 × 10^8^	5.34 × 10^11^	3760

### Sediment yield and flux estimates for the paleo Bindschadler Ice Stream

Detailed studies of other glaciated and formerly glaciated margins show that erosion rates (i.e., yield) are primarily dependent on the glacierization mode (e.g., a temperate versus polar glacier)^14^. The nature of the substrate is also important^15^. The highest erosion rates are associated with fast flowing Alaskan valley glaciers flowing over fractured bedrock and the lowest erosion rates are associated with slow-flowing dry polar glaciers^15^. Several studies have strongly suggested that paleo sediment yield is also dependent upon whether grounded ice is in the advance-mode or retreat-mode of a large-scale grounding-line-translation cycle^16–18^. Lower yield during gradual glacial advance is attributed to the slow flow and comparatively little meltwater whereas higher sediment yield during deglaciation is associated with faster ice motion. The available age control indicates that the outer shelf CGZW in the Whales Deep Basin formed during the early deglacial^4^. Unfortunately, there are no data-based estimates of early-deglacial paleo sediment yield and flux for WAIS ice streams catchments. For this reason, we turn to the modern ice streams. Alley *et al*.^19, 20^ estimated that the catchments of modern WAIS ice streams have an average erosion rates of 0.4–0.5 mma^−1^. Whereas more recent observations indicate that these older estimates of till flux and subglacial erosion may err on the high side^21^, we still use these potentially too high erosion rates because our primary goal is to produce a lower bound duration of CGZW formation. Ice flow during the early deglacial may have been faster and more continuous as the WAIS experienced a prolonged negative mass balance during this timeframe. Elverhoi *et al*.^14^ suggested that early deglacial sediment yield may have been 25% higher than the longer term deglacial erosion rates as ice streamed over the unconsolidated sediment recently deposited during the preceding advance of grounded ice. Modeling studies also use the assumption that paleo ice-stream sediment flux and erosion rates were higher during the early interglacial^22^. To account for the possibility that sediment yield during the early deglacial was higher than the average modern yield, we utilized an upper bound paleo sediment yield estimate of 0.625 mma^−1^ for the paleo Bindschadler Ice Stream. Extrapolation of this upper-bound paleo sediment yield (i.e., 0.625 mma^−1^) over the larger paleo drainage area results in a paleo sediment flux, *Q*_P_, of 1.42 × 10^8^ m^3^a^−1^ (Table 1). Using Equation (2), we estimate that the duration of the outer-shelf grounding events was 3760 ± 247 years (Table 1). The error for the grounding-duration estimate includes a ±2 m uncertainty associated with the TWTT measurements of the CGZW’s upper and lower bounding seismic reflections. The error also includes ±50 ms^−1^ uncertainty in the velocity used to convert the isopach from TWTT to depth.

## Discussion

### **L**argest CGZW yet mapped in Antarctica

The Whales Deep CGZW clearly correspond to a time when the grounding line occupied several positions while the WAIS was grounded on the outer shelf and drained a large area of central West Antarctica. The lower reach of the drainage basin is underlain by unconsolidated sedimentary strata of upper Cenozoic age^23^. The upper-most reaches are deeply excavated and exhibit high relief (Fig. 1) suggesting basement exposure based on comparison with other regions^24^. This large interior region and the trough axis was undoubtedly the source material for the outer-shelf GZWs.

The outer shelf CGZW volume is greater than the large GZWs in Joides, Drygalski and Glomar Challenger Basins that have been seismically mapped in the western and central Ross embayment^12, 25–27^. Reflections that bound the GZWs are not regional in extent, and hence, the volumes of the individual GZWs cannot be estimated. It is difficult to directly compare the Whales Deep CGZW volume to other GZW systems that have been surveyed with the coarser-spaced seismic and multibeam data^24–31^ because the published data cannot be used to generate robust estimates of the unit’s total dimensions. Given that other GZWs are confined to relatively narrow paleo-ice-stream troughs (<50-km widths), the outer-shelf CGZW in Whales Deep is the largest system yet mapped and thus likely records a relatively prolonged grounding.

### An inferred post-LGM age of the Whales Deep CGZW

The stratal stacking of the CGZW permits that the wedges may have formed during discrete cycles of WAIS advance and retreat that so happened to reach a similar grounding line position during successive glacial periods. Here we show that the CGZW is a post-LGM feature. The Whales Deep outer shelf CGZW (Fig. 3) corresponds to GZW5b from Mosola and Anderson^4^. Stratigraphic superposition requires that GZW5b is younger than GZW5a, the wedge of sediment that underlies the outer shelf and upper slope (Fig. 3). Cores collected from the outer shelf by Mosola and Anderson^4^ penetrated subglacial till from GZW5a at core station 13 (Fig. 3). At this station, four radiocarbon dates were obtained from the post-glacial sediment that overly the subglacial tills. These and the other radiocarbon dates from the basin (Fig. 3) were derived exclusively from bulk acid insoluble organic matter (AIOM). The ages are considered suspect because bulk AIOM contains some unknown fraction of older carbon reworked from strata exposed in the Whales Deep paleo drainage basin mixed with the *in situ* organic carbon that was contemporaneously deposited with the deglacial marine sediment. Hence, the mixing of recycled and contemporaneously produced carbon yields ages that are older than actual. The age offset depends upon the relative proportions of recycled and *in situ* carbon and unfortunately, the mix ratios are likely to have been variable thru time^32^. Within the context of these limitations, the actual dates at which marine sedimentation resumed had to have occurred after the time indicated by the oldest bulk AIOM radiocarbon date from deglacial sediment. Based on the oldest dates from core station 13, grounded ice moved south of this location and marine sedimentation resumed at some time after 23,907 +/− 300 calibrated ^14^C yrs. Hence, by stratigraphic superposition, the overlying CGZW had to have been deposited within less than 23,907 +/− 300 years. An additional constraint on the CGZW duration comes from Conway *et al*.^33^ who showed that grounded ice moved south of Roosevelt Island by 3,200 years. By these constraints, the maximum possible duration of the CGZW is taken to be >20,000 years. This stratal arrangement precludes the possibility that the outer shelf CGZW represents the deposits associated with glacial cycles that predate the LGM.

### Paleo Bindschadler Ice Stream sediment yield and flux

Sediment yield and flux from the paleo Bindschadler Ice Stream catchment would have been dependent upon many factors (including but not limited to sediment type, substrate fracturing, ice stream velocity, and presence/quantity of meltwater). Early studies emphasized deforming till layers, which were proposed to range in thickness from 2 to 15 m. Alley *et al*.^19^ proposed that the deforming till layer velocity decreases with depth. More recent studies suggest that that fast flow is confined to a narrow zone. Decreasing porosity with depth in the till layer suggested to Kamb^2^ that only the upper few centimeters of the subglacial till layer may experience fast flow. Thus, borehole observations of till velocity for the Bindschadler ice core, pertain only to the upper few centimeters of till flux into which a tethered stake was set^2^. To date, only one study of an Alaskan valley glacier has demonstrated that a considerable thickness of deformation till (the upper 2 m of a 7 m basal till layer) experiences fast plug-like flow^34^. In addition to sediment transport in a deforming basal till layer, recent studies indicate that a significant volume of sediment could also be transported to the grounding line in basal ice layers^35^. Melting at the grounding line demonstrates that a significant volume of sediment transported in basal ice layers could be deposited at the grounding line^36^. Hence, the paleo sediment flux across the grounding line depends on the combined contribution of deformation till and sediment concentrated in basal ice layers. We assume that the relatively high erosion rate of 0.625 mma^−1^ used here reflects an upper bound input of all sedimentary material to the CGZW, whether transported to the paleo-grounding line subglacially or in the basal ice. The width of the paleo Bindschadler Ice Stream on the outer shelf is ~100,000 m (Fig. 1). A paleo sediment flux of 1.42 × 10^8^ m^3^a^−1^ could have been associated with several combinations of cumulative vertical thickness of sediment (from both deformation till and sediment-rich basal-ice-layer) and net velocity (Table 2). If sediment deposited in the CGZW were exclusively derived from a deforming basal till layer, than only fast-flowing thin sediment layers would be consistent with borehole observational data^2^.

**Table 2:** (A) Paleo Bindschadler Ice Stream sediment flux; (B) Paleo Bindschadler Ice Stream width.

A) Paleo-BIS sediment flux *Q*_p_ (in m^3^a^−1^)	B) Paleo-BIS width (in meters)	C) Sediment- layer* thickness (in meters)	D) Sediment-layer* velocity (in ma^−1^)
1.42 × 10^8^	100000	4.7	300
		2.8	500
		1.4	1000
		0.94	1500
		0.7	2000

(C–D) Possible pairs of effective sediment layer thickness (from both deforming basal till and sediment-rich basal ice layer) and sediment-layer velocity, respectively. *The sediment layer includes the deformation till and cumulative sediment within basal-ice layers. BIS = Bindschadler Ice Stream.

Our upper bound estimate for the paleo sediment yield (0.625 mma^−1^) for the early deglacial is consistent with the view that 1) the overall mode of glacierization did not change significantly between the early deglacial and the modern and that 2) the early deglacial flux should be slightly higher^14, 22^ relative to that estimated by Alley *et al*.^19^. Our use of a 0.625 mma^−1^ yield as a representative paleo yield for the early deglacial is also supported by a comparison to paleo sediment yields estimated for the similarly wide and long ice streams that drained large-drainage basins for the marine-based ice sheet that formerly covered Barents and North Seas. In those areas, erosion rates averaged ~1 mma^−1^ after the peak of the LGM^37^. Our paleo flux estimate for the paleo Bindschadler Ice Stream (1.4 × 10^8^ m^3^a^−1^) is of the same order but slightly lower than that for the Norwegian Channel Ice Stream^37^. At that time, grounded ice advanced to the North Sea shelf edge^38^, the Norwegian Channel drained an area from 6.1 to 7.8 × 10^11^ m^2^. Hence, the greater sediment flux values for the Norwegian Channel ice stream was due to a combination of a larger drainage area and a higher average yield (i.e., 1 mma^−1^). Even higher yields of 2.4 mma^−1^ were associated with the Storfjorden and Bear Island ice streams in the middle Pleistocene^14^. Those high yields were attributed to the onset of large amplitude oscillations of grounding line across previously unglaciated landscapes mantled by unconsolidated sediment cover. Modern yields from small temperate Alaskan valley glaciers and those in the Swiss Alps are the highest reported erosion rates (5–10 mma^−1^) but these rates are due to high relief, erodible rock types, and highly fractured substrates in the presence of voluminous melt water^15^. We do not favor the view that paleo sediment yields could have exceeded 1 mma^−1^ in the Whales Deep drainage area because such high values are more typical for temperate mountain glaciers flowing over high relief and fractured substrates in settings such as Alaska^15, 39^ whereas the WAIS drainage area is relatively flat and presumably consolidated Cenozoic rock^40^. Relatively low long-term rates of sub-ice stream erosion in the Ross Sea sector of WAIS are also supported by the fact that these ice streams are associated with troughs that have relatively low relief. In contrast, the very high erosion rates beneath temperate mountain glaciers produce valleys with topographic relief that is one to two orders of magnitude greater than observed in our study region.

### A long grounding and an abrupt retreat

Our calculation provides a reasonable estimate of grounding duration. However, it is important to note that previous studies suggest that the vertical thickness and horizontal width of the deformation till layer are likely to vary over time and from ice stream to ice stream^2, 41^. Moreover, our paleo sediment flux estimate assumed continuous ice streaming but fast flow may also have intermittently been dominated by basal sliding^41^. Intervals of ice-stream deceleration, stagnation or shift to streaming by basal sliding would all cause sediment flux to diminish or cease. In this sense, our sediment flux might be considered a maximum and hence the estimated duration a minimum. However, even this minimum is a relatively long period grounding, as ca. 3700 years represents about one third of the total time that it took for the WAIS grounding line in the Ross Sea sector to retreat from its LGM maximum to near the present-day position^33^.

There is no definitive explanation as to why the WAIS grounding line paused so long on the outer shelf in the Whales Deep Basin. However, neither the duration nor the available age control is sufficiently resolved to establish a correlation to post-LGM sea level changes or other possible mechanisms^42^. Bathymetric controls, such as constrictions in troughs and bathymetric highs, have been proposed a mechanism for stabilizing grounding zones^43–45^. In the case of Whales Deep, there are no nearby shallow pinning points or a significant bottleneck in the trough (at the level of the unconformity underlying the CGZW) created by the orientation of the Hayes and Houtz banks. At the Whales Deep outer shelf, the overall >100 m aggradation of sediment at the grounding line is consistent with previous conjecture that sedimentation at the grounding line itself exerts a control on grounding line stability^46^. Hence, the CGZW may be an example of a situation where such depositional processes created and maintained a pinning point, which stabilized the regional grounding line position for thousands of years.

A 3700-year outer shelf grounding is significant because it provides perspective as to the possible durations of past and modern stationary grounding line positions and the presumably stable ice sheet mass balance. In comparison, GZWs elsewhere on the Antarctic margins with smaller volumes have been inferred to represent significantly shorter duration grounding events ranging from a few decades to slightly longer than a millennium^31, 43, 47^. The long grounding is also significant with respect to how such a long interval with a stationary grounding line was followed by geologically instantaneous long distance retreat of grounded ice from the outer shelf to Roosevelt Island. The rapid retreat phase may represent compensation for the fact that GZW growth phase makes an ice stream grounding line stable despite climate and sea level forcings. Once those factors make grounding at the GZW untenable, then the resulting retreat to a new stable position is large and rapid. Although the exact radiometric timing and duration is yet to be determined, the data clearly highlight an important paradoxical duality to the WAIS dynamics with long intervals of relatively stationary grounding lines followed by rapid return of grounded ice to the global ocean.

## Conclusions

Our estimate of sediment flux for the paleo Bindschadler Ice Stream predicts that the CGZW would have taken on the order of 3700 years to deposit. The absence of any seismically resolvable GZWs south of the bathymetric saddle indicates that a large distance retreat of the grounding line from the outer shelf to the latitude of Roosevelt Island occurred in a geologic instant. The long-duration grounding followed by an abrupt large-distance grounding line retreat may have resulted from a highly non-linear ice sheet response to relatively continuous external forcing such as gradual climate warming or sea level rise. Our findings indicate that reliable predictions of future WAIS retreat may require incorporation of realistic calculations of sediment erosion, transport and deposition.

## Electronic supplementary material

Supplemental Figure
