# Supplementary material for: The paradox of a long grounding during West Antarctic Ice Sheet retreat in Ross Sea
Source: Sci Rep. 2017 Apr 28;7:1262. doi: 10.1038/s41598-017-01329-8 (PMC5430838; doi:10.1038/s41598-017-01329-8)
Supplement: Supplementary file 1 — Supplemental Figure [file 41598_2017_1329_MOESM1_ESM.pdf]

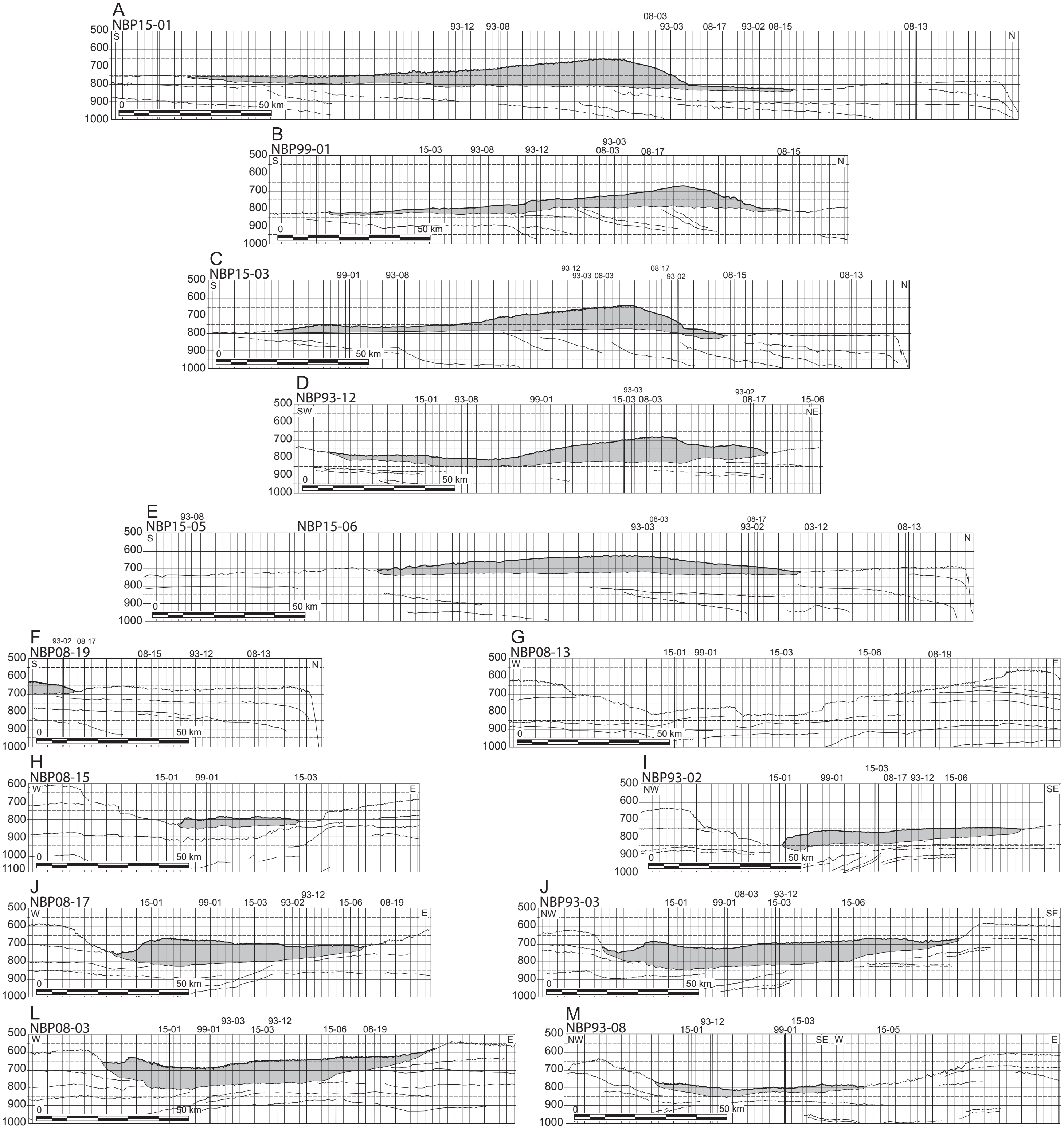

From SREP-16-40884A
The paradox of a two millennia grounding duration of the West Antarctic Ice Sheet in Ross Sea
Philip J. Bart* Benjamin J. Krogmeier, Manon P. Bart and Slawek Tulaczyk
